# TGFβ1 induces hypertrophic change and expression of angiogenic factors in human chondrocytes

**DOI:** 10.18632/oncotarget.20509

**Published:** 2017-08-24

**Authors:** Jie-Lin Chen, Chang Zou, Yunfang Chen, Weimin Zhu, Wei Liu, Jianghong Huang, Qisong Liu, Daming Wang, Li Duan, Jianyi Xiong, Jiaming Cui, Zhaofeng Jia, Daping Wang

**Affiliations:** ^1^ Shenzhen Key Laboratory of Tissue Engineering, Shenzhen Second People’s Hospital, The First Affiliated Hospital of Shenzhen University, Shenzhen 518035, Guangdong Province, China; ^2^ Shenzhen Centre for Sports Medicine and Engineering Technology, Shenzhen 518035, Guangdong Province, China; ^3^ Shenzhen Public Service Platform for Cancer Precision Medicine and Molecular Diagnosis, Shenzhen 518020, China; ^4^ Clinical Medical Research Center, The Second Clinical Medical College, Shenzhen People’s Hospital, Jinan University, Shenzhen, 518020 China; ^5^ The Eighth Affiliated Hospital, Sun Yat-sen University, Shenzhen 518035, Guangdong Province, China

**Keywords:** TGFβ1, chondrocyte, hypertrophy, angiogenesis, DNA microarray

## Abstract

The transforming growth factor β1 (TGFβ1) plays an important role in cartilage development. However, whether TGFβ1 stimulates chondrocyte proliferation and cartilage regeneration in osteoarthritis (OA) remains elusive, especially in the context of different treatment and tissue environments. In the present study, we investigated the role of TGFβ1 in human chondrocyte culture *in vitro*, focusing on the morphological change of chondrocytes and the expression of angiogenic factors upon TGFβ1 stimulation. We found increased expression of biomarkers indicating chondrocyte hypertrophy and the chondrocytes aggregated to form networks when they were treated with TGFβ1. DNA microarray analysis revealed significantly increased expression of genes related to blood vessel formation in TGFβ1 treatment group compared to control group. Matrigel assay further demonstrated that chondrocytes had the potential to form network-like structure. These results suggested that TGFβ1 induces the hypertrophic change of chondrocytes culture *in vitro* and induce expression of angiogenic biomarkers. Therefore, application of TGFβ1 for chondrocyte culture in practice should be considered prudentially and targeting TGFβ1 or relevant receptors to block the signaling pathway might be a strategy to prevent or alleviate progression of osteoarthritis.

## INTRODUCTION

Osteoarthritis (OA) is a common degenerative disorder which affects the function of joints, causes pains and reduces quality of life in patients. In the pathogenesis of osteoarthritis, chondrocytes in the cartilage undergo hypertrophic change and the cartilage-specific extracellular matrix (ECM) gradually degrades. Blood vessels and nerves invade the cartilage thereafter, exacerbating the condition of affected joint [1]. Currently, there is no identified cause of blood vessel invasion and effective strategy for the treatment of osteoarthritis [2].

Increased angiogenic factors and/or decreased anti-angiogenic factors promote blood vessel formation [3]. Although there is no blood vessel in normal cartilage, there are angiogenic factors, such as fibroblast growth factor (FGF) and vascular endothelial growth factor (VEGF), arising from synovium and subchondral bone [4]. Therefore, cartilage possesses the potential of angiogenesis. However, the capability of angiogenesis in cartilage may be inhibited by anti-angiogenic factors in the microenvironment, which helps maintain the characteristics of healthy cartilage. Once the balance is broken, excessive angiogenic factors will promote blood vessel formation, as it was evidenced in inflammatory conditions [5]. As blood vessels play a devastating role in osteoarthritis, inhibition of angiogenesisis a strategy for the treatment. Therefore, elucidating the mechanisms of angiogenesis in osteoarthritis will be of great importance [6].

Transforming growth factor β1 (TGFβ1) has been investigated in osteoarthritis and chondrocytes for years [7, 8]. However, reports of its function varied in published data from different laboratories [9–11]. Previously, most studies reported that TGFβ1 was essential for cartilage integrity and ECM maintenance. Blockage of TGFβ1 signaling resulted in cartilage degeneration and osteoarthritis-like tissue formation [7]. More recent studies have demonstrated that TGFβ1 might accelerate the degeneration of cartilage, including hypertrophy, angiogenesis and ECM mineralization [12]. In patients with osteoarthritis, there was high level of TGFβ1 in subchondral bone and inhibition of the activity of TGFβ1 effectively attenuated the degradation of cartilage ECM, suggesting that high level of TGFβ1 in subchondral bone be a trigger of osteoarthritis [13, 14]. Further studies revealed that TGFβ1 could induce the aggregation of mesenchymal stem cells (MSC) and induce the blood vessel formation in osteoarthritis cartilage [13]. However, whether TGFβ1 induces morphological change and expression of angiogenic factors in chondrocytes cultured *in vitro* and the corresponding mechanisms were not identified [15].

In the present study, we investigated the effect of TGFβ1 on human chondrocytes cultured *in vitro*. DNA microarray was performed to screen the differentially expressed genes (DEGs) in chondrocytes culture. It was found that genes involved in chondrocyte hypertrophy and blood vessel development were significantly upregulated in TGFβ1 treated chondrocytes. Our results demonstrated that human chondrocytes treated with TGFβ1 *in vitro* undergo hypertrophic change and be induced to express angiogenic factors.

## RESULTS

### Isolation and identification of human articular chondrocytes

**Figure 1:**
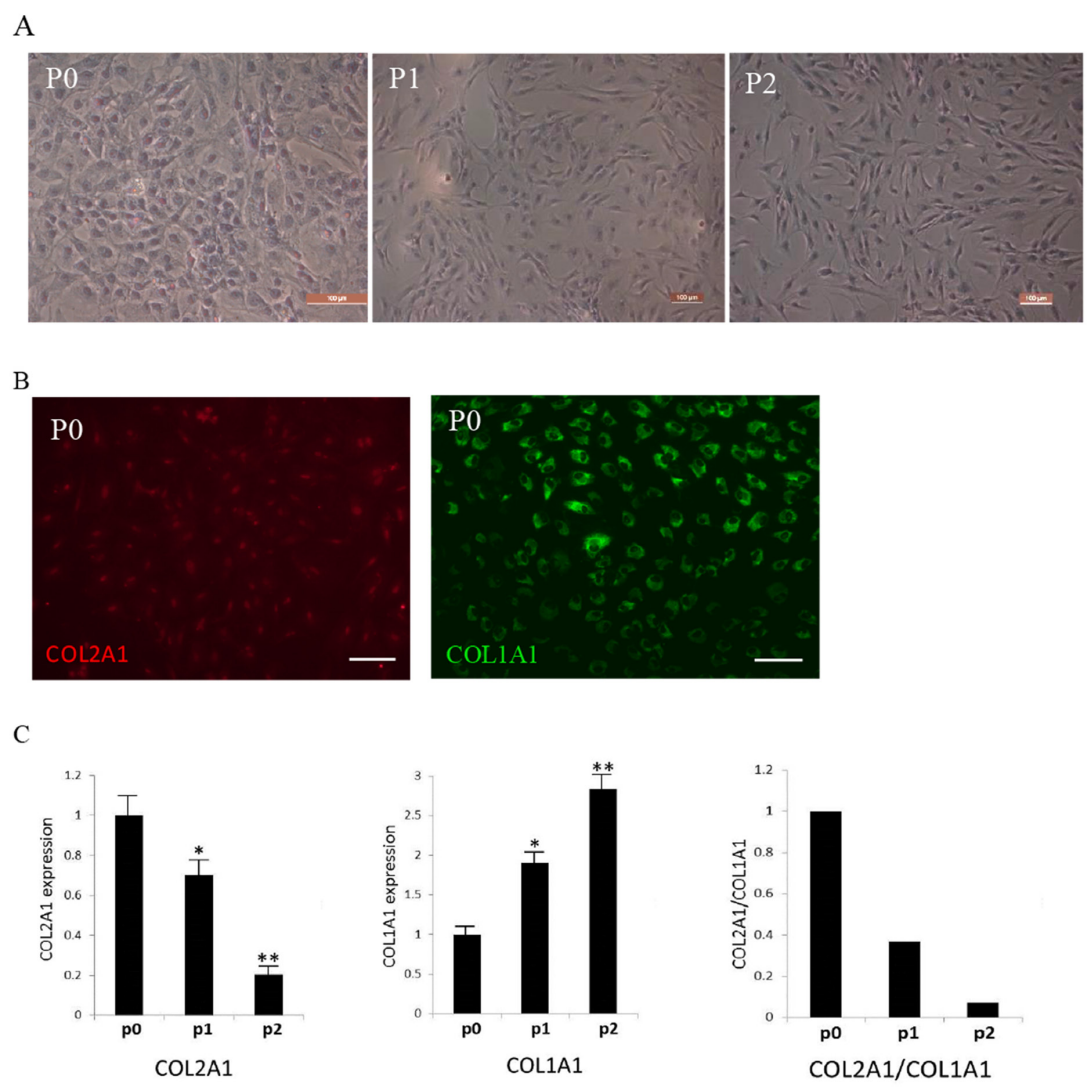
Identification of isolated human articular chondrocytes **(A)** Toluidine blue staining (TB) of P0-P2 chondrocytes. **(B)** P0 chondrocytes were immunostained with type I (COL1A1) and type II collagen (COL2A1). **(C)** Examination of COL2A1 and COL1A1 expression by quantitative RT-PCR. ^*^ significant difference in gene expression (^**^P<0.01 and ^*^P<0.05).Scale bar: 100μm.

Collagen type II, type I and glycosaminoglycan (GAG) were the primary components in the cartilage ECM and were used as the criteria for the identification and evaluation of chondrocytes [9, 18]. Human articular chondrocytes were isolated and evaluated with toluidine blue staining, immunocytochemical staining and quantitative RT-PCR. Toluidine blue staining revealed production of GAGs decreased in -passage 1(P1) and passage 2(P2) cells compared with primary P0 cells (Figure 1A). In immunocytochemical staining, Col2A1 was detected only in P0 chondrocytes as the signals were very weak in chondrocytes of P1 and later passages (Figure 1B). Col1A1 was detected in all passages (Figure 1B). Quantitative RT-PCR demonstrated a decreased expression of Col2A1 and an increased expression of Col1A1 during cell passaging (Figure 1C). These results suggested that chondrocytes underwent dedifferentiation during passaging.

### Cells aggregated upon TGFβ1 treatment

**Figure 2:**
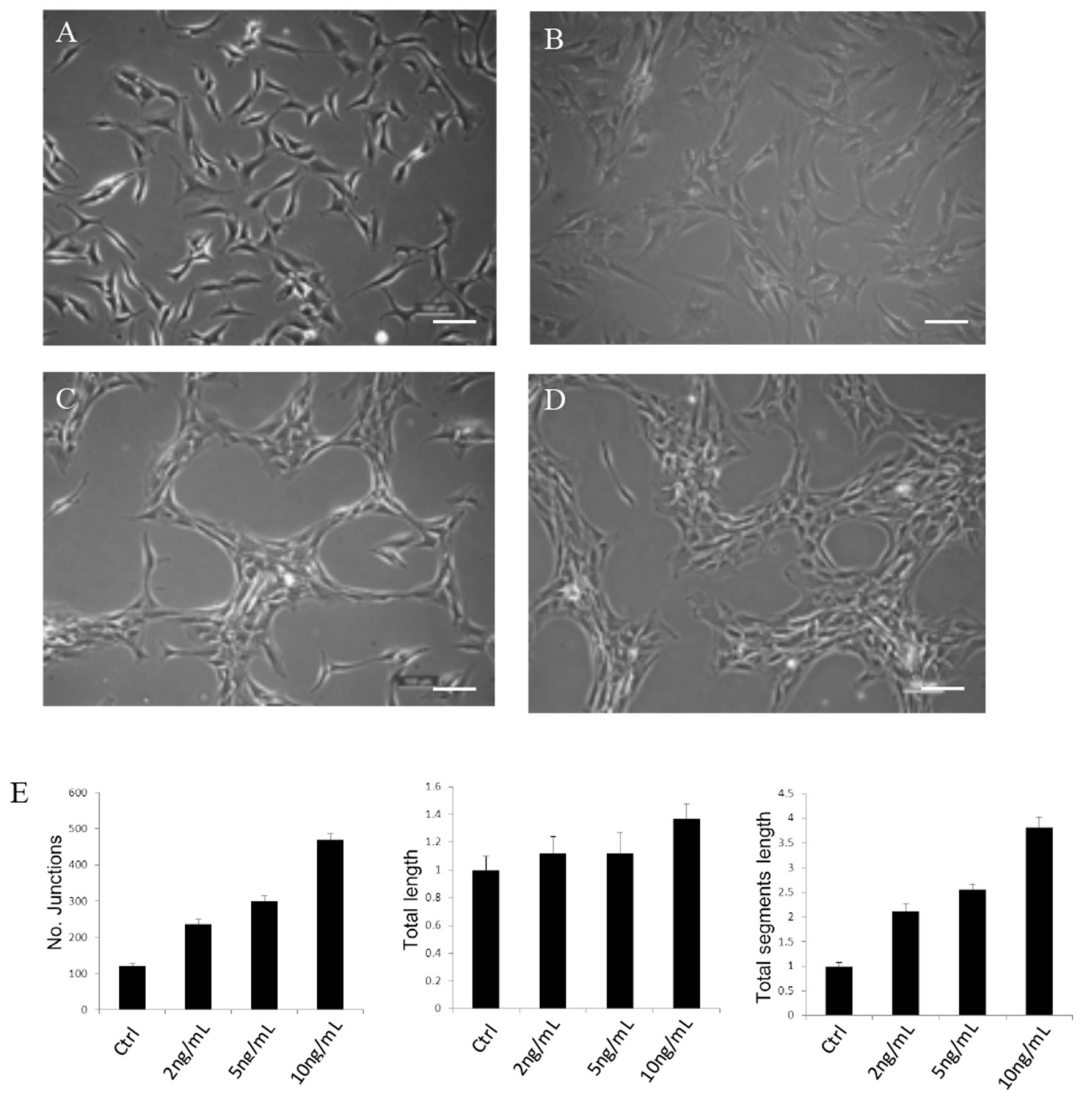
Representative cell images of chondrocytes taken at day 3 treated without TGFβ1 **(A)** and with 2ng/ml **(B)** 5ng/ml **(C)** 10ng/ml **(D)** TGFβ1 and quantitative analysis of network formation. Images shown are representative of at least three separate experiments. Cells started to aggregate after treatment with TGFβ1 for 1 day and form network-like structure in about 3 days. A concentration of more than 2ng/ml was high enough to induce network formation. Scale bar: 100μm.

**Figure 3:**
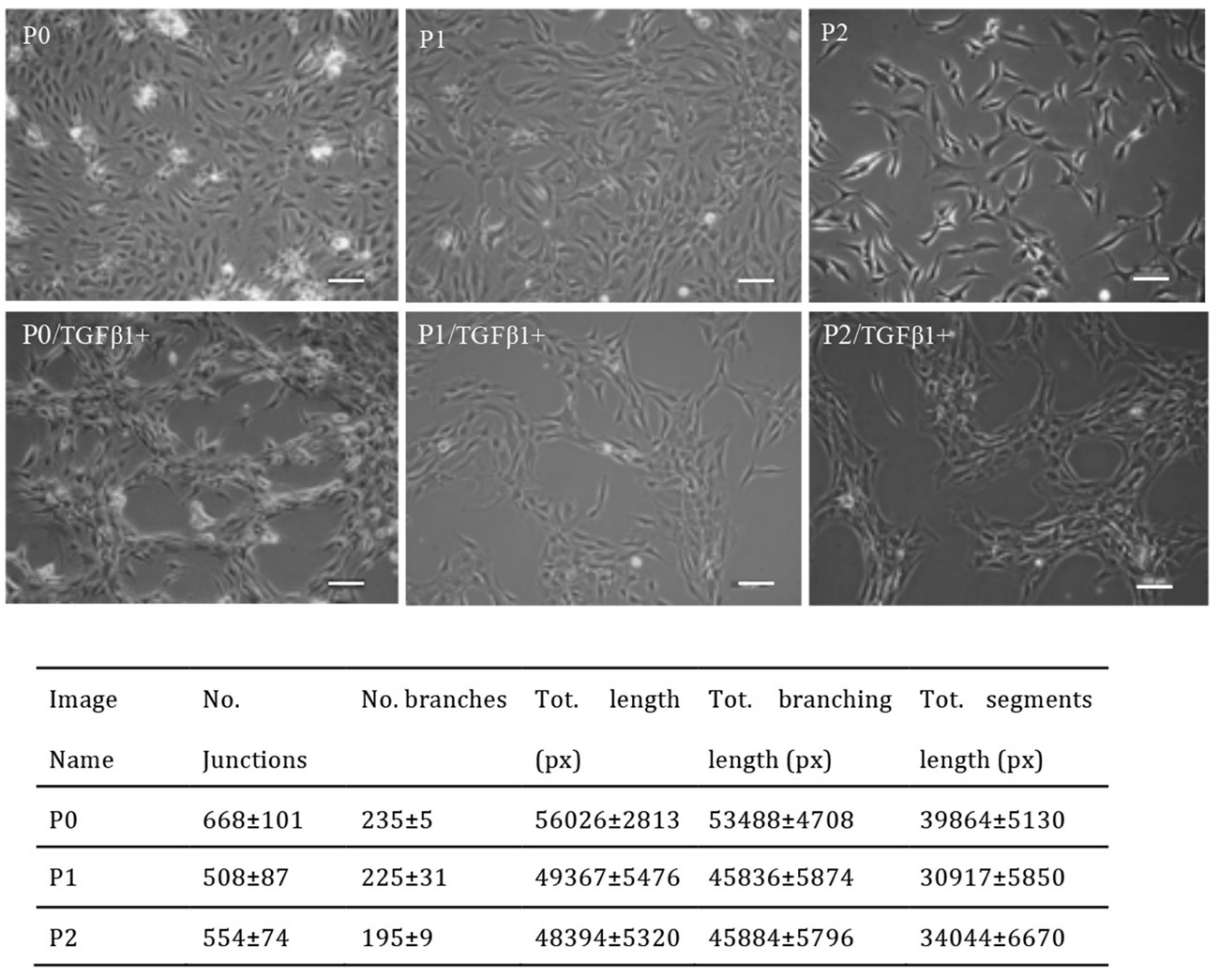
Representative images of P0 to P2 chondrocytes treated with 10ng/ml TGFβ1 and quantitative analysis of network formation Images shown are representative of at least three separate experiments. Cells from P0 to P2 started to aggregate after treatment with TGFβ1 for 1 day and form network in 3 days. Scale bar: 100μm.

Generally, chondrocytes cultured *in vitro* were evenly distributed on the plate (Figure 2A). Upon TGFβ1 treatment, cells moved and aggregated, forming network-like structure after 3 days in culture (Figure 2B-2D). Different concentrations of TGFβ1 have been used in the experiment. A concentration of more than 2ng/ml was high enough to promote chondrocytes to aggregate. The number of junctions, total length and total segment length increased with increasing TGFβ1 concentrations. Additionally, chondrocytes of P0 to P2 passages formed network-like structure with TGFβ1 treatment and data of the P0 to P2 were very close, with P0 slightly higher. Representative images of chondrocytes from P0 to P2 treated with TGFβ1 were shown in Figure 3.

### Differentially expressed genes screened by DNA microarray analysis

A total of 2416 differentially expressed genes (fold change ≥ 5 and fold change≤ 0.2) were found in 10ng/mL TGFβ1-treated chondrocytes. Of these genes, 956 genes were up-regulated and 1450 genes were down-regulated. The top 10 most significantly up- or down-regulated DEGs were listed in Table 1. These genes were involved in cell adhesion and movement (AMIGO2, CLEC18A, CDH2, SEPP1 and ITGB8), cell growth and differentiation (CDKN2B, IL11, TSPAN2 and NTRK2), cellular metabolism (ST6GAL2, STC1, SERPINE1 and CFD), oxidation-reduction process (NOX4,FMO2,FMO3 and AKR1C1) and development (COL10A1, MEST and FOXQ1). According to Gene Ontology (GO) analysis, the DEGs were enriched in system development, blood vessel development, regulation of signaling, response to external stimulus and functions listed in Table 2. KEGG analysis revealed the genes could be classified to gap junction, ECM-receptor interaction and MAPK signaling pathways in Table 3. As expected, TGFβ1 signaling pathway was also detected.

**Table 1:** The top 10 most significantly up- or down-regulated DEGs

Gene ID	Gene Symbol	Gene Title	Fold Change
Up-regulated genes			
AW004016	ST6GAL2	ST6 beta-galactosamide alpha-2,6-sialyltranferase 2	79.4
AI376003	COL10A1	collagen type X alpha 1	75.9
AC004010	AMIGO2	adhesion molecule with Ig-like domain 2	61.7
AW444761	CDKN2B	cyclin-dependent kinase inhibitor 2B	32.6
BF057185	CLEC18A	C-type lectin domain family 18, member A	31
NM_000641.1	IL11	interleukin 11	30.3
NM_016931.1	NOX4	NADPH oxidase 4	24.4
BF129969	TSPAN2	tetraspanin 2	16.7
M34064.1	CDH2	cadherin 2, type 1, N-cadherin	12.3
BC020765.1	SERPINE1	serpin peptidase inhibitor, clade E	9.9
Down-regulated genes			
NM_005410	SEPP1	selenoprotein P, plasma, 1	0.013
AI758223	FMO2	flavin containing monooxygenase 2	0.021
BF513121	ITGB8	integrin, beta 8	0.024
AA707199	NTRK2	neurotrophic tyrosine kinase, receptor, type 2	0.025
NM_003155	STC1	stanniocalcin 1	0.033
NM_002402	MEST	mesoderm specific transcript homolog	0.037
AI676059	FOXQ1	forkhead box Q1	0.042
M83772	FMO3	flavin containing monooxygenase 3	0.044
BF508244	AKR1C1	secreted frizzled-related protein 1	0.049
NM_001928	CFD	complement factor D	0.05

**Table 2:** The enriched GO functions of DEGs

Names	Genes_In_Term	DEG	Significant
GO:0048731//system development	6697	781	TRUE
GO:0048523//negative regulation of cellular process	6308	734	TRUE
GO:0007275//multicellular organismal development	7875	890	TRUE
GO:0048519//negative regulation of biological process	6805	783	TRUE
GO:0009966//regulation of signal transduction	4170	512	TRUE
GO:0032502//developmental process	8833	979	TRUE
GO:0001568//blood vessel development	891	146	TRUE
GO:0048856//anatomical structure development	7770	873	TRUE
GO:0001944//vasculature development	950	152	TRUE
GO:0044699//single-organism process	21723	2173	TRUE
GO:0009605//response to external stimulus	2033	276	TRUE
GO:0010646//regulation of cell communication	4670	556	TRUE
GO:0023051//regulation of signaling	4645	553	TRUE

Guide: Names-pathways, Genes in Term- number of genes in the term, DEG- number of differentially expressed genes.

**Table 3:** The enriched KEGG pathways of DEGs

Names		Genes_In_Term	DEG	Significant
Total		20594	1788	-
ko04540	Gap junction	315	55	TRUE
ko04350	TGF-beta signaling pathway	244	41	TRUE
ko04512	ECM-receptor interaction	338	52	TRUE
ko04115	p53 signaling pathway	250	41	TRUE
ko00380	Tryptophan metabolism	123	23	TRUE
ko00910	Nitrogen metabolism	72	15	TRUE
ko03030	DNA replication	87	17	TRUE
ko04510	Focal adhesion	898	104	TRUE
ko04010	MAPK signaling pathway	907	104	TRUE

Note: Names-pathways, Genes inTerm-number of genes in the term, DEG-number of differentially expressed genes.

### Gene expression validation

**Figure 4:**
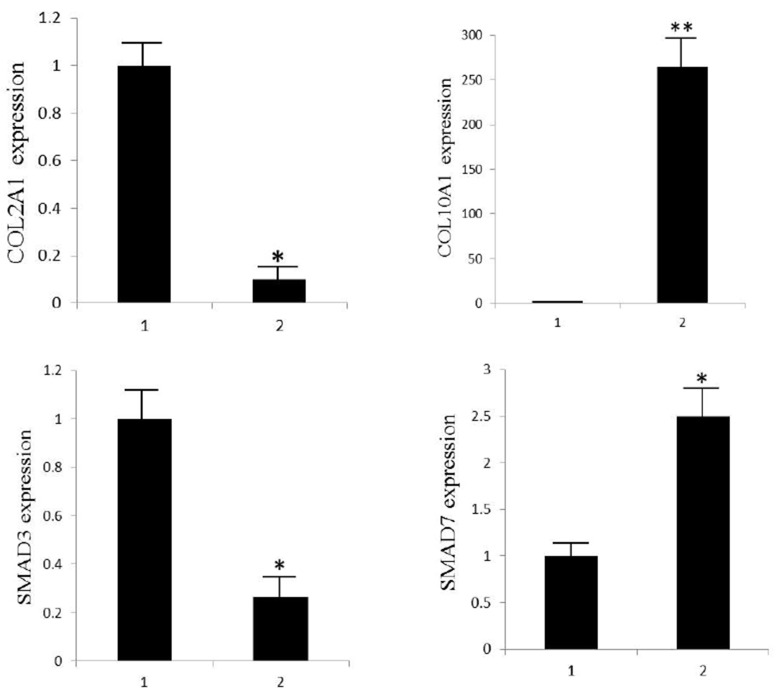
Quantitative RT-PCR analysis of COL2A1, COL10A1, SMAD3 and SMAD7 in TGFβ1 treated and control groups Transcript levels were assessed on three different cultures; histograms depict average levels; error bars depict standard deviation values. ^*^significant difference in gene expression (^**^P<0.01 and ^*^P<0.05).

Genes associated with cellular hypertrophy and in TGFβ signaling pathway were examined by quantitative RT-PCR (Figure 4). Expression of COL2A1 was significantly decreased while COL10A1 drastically increased, suggesting the cells undergo hypertrophic change during the culture. As important molecules in TGFβ signaling pathway, SMAD3 was down-regulated while the inhibitory molecule SMAD7 was up-regulated, which was in consistent with studies by Narcisi R and Yang YH [15, 19].

**Figure 5:**
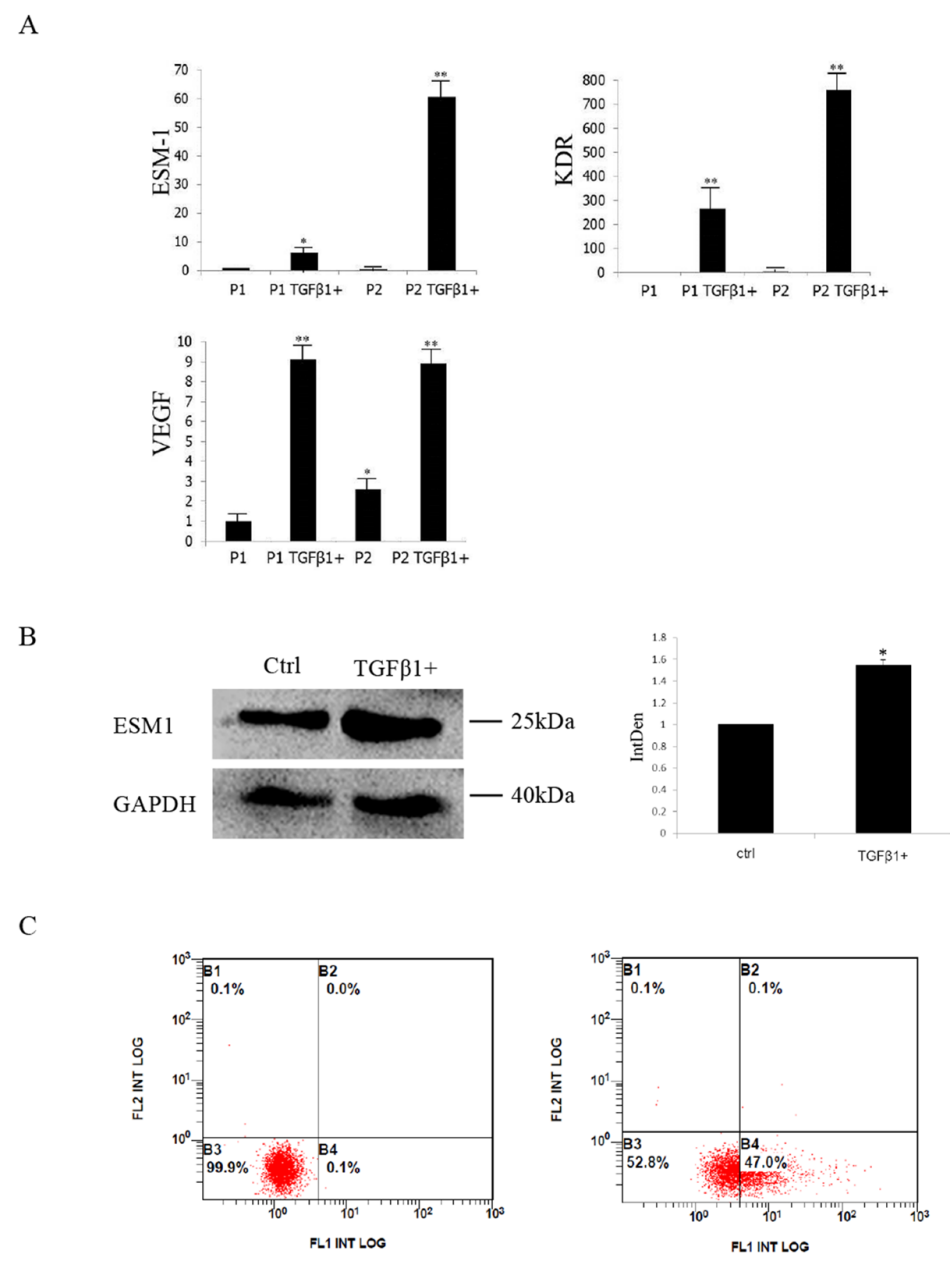
Differentially expressed genes studied in microarray experiments were validated by qRT-PCR, Western blot and Flow Cytometric analysis **(A)** Quantitative RT-PCR analysis of ESM1, KDR and VEGF in 10ng/ml TGFβ1 treated and control groups. **(B)** ESM1 validation by Western blot; GAPDH was used as the internal control. **(C)** ESM1 validation by Flow Cytometric analysis. ESM1 positive cells increased after TGFβ1 treatment. ^*^ significant difference in gene expression (^**^P<0.01 and ^*^P<0.05).

Microarray-screened DEGs involved in blood vessel formation were also examined. Endothelial cell-specific molecule 1 (ESM1), vascular endothelial growth factor receptor 2 (KDR/VEGFR2) and VEGF were selected and validated. In consistent with microarray analysis, expression of all the three genes increased significantly (Figure 5A). ESM1 was further validated by Western blot and Flow Cytometric analysis (Figure 5B–5C), which demonstrated significant increase at the protein level, with 1.53 fold by Western blot analysis.

### Network formation of chondrocytes in matrigel

**Figure 6:**
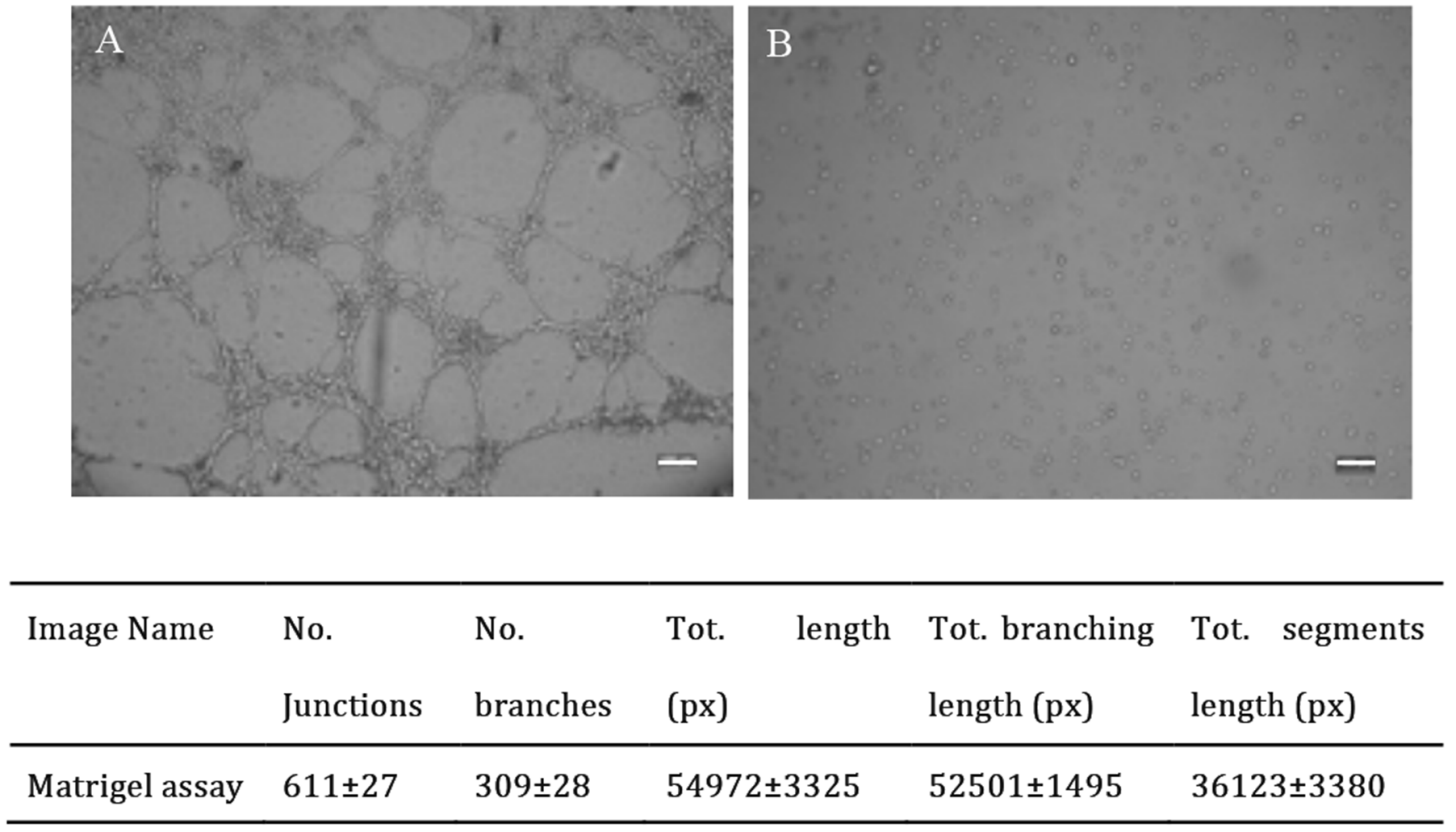
Representative images and quantitative analysis of network formation by chondrocytes in Matrigel assay P1 chondrocytes seeded in the matrigel formed networks after 5 hours in culture **(A)** No network was observed in the wells with media plus angiogenesis inhibitor **(B)** Scale bar: 100μm

As genes enriched in blood vessel formation were validated and chondrocytes had the capability to form networks in monolayer culture, the cells were then used for a matrigel assay to observe whether the cells could form networks in the gel. As the results showed, cell networks were observed from 2 out of 7 samples (Figure 6). No network formed in the control group.

## DISCUSSION

The TGFβ signaling pathway has been investigated in osteoarthritis and cartilage, but its effects on chondrocyte proliferation and cartilage ECM production were controversial [9, 20, 21]. TGFβ1 can promote upregulation of collagen and prevent loss of proteoglycan in articular cartilage [22, 23]. However, exogenous TGFβ administration induced osteoarthritis-like changes of the cartilage [24]. In the present study, the role of TGFβ1 in human chondrocytes cultured *in vitro* was investigated.

From our observations, chondrocytes treated with TGFβ1 aggregated with each other, forming network-like structure in the culture plate. Elevated expression of COL10A1 and decreased level of COL2A1 suggested that chondrocytes underwent hypertrophic change, which was in consistent with the research by R.NARCISI [15]. Upon microarray analysis, increased expression of angiogenic factors were detected from the chondrocytes treated with TGFβ1. Surprisingly, cells from 2 out of 7 samples formed network-like structure in matrigel assay. The formation of cell networks might be due to angiogenic factors, endothelial cells or precursor cells in the sample. Another explanation might be the chondrocytes underwent trans-differentiation to endothelial cells after TGFβ1 treatment as it was shown endothelial cells could trans-differentiate to chondrocyte-like cells under high-glucose circumstances [25]. Identification of MSC markers or chondrocyte markers would determine whether the cells underwent trans-differentiation [25, 26]. As cells from 5 out of 7 samples did not form networks in the gel, more investigations are needed. Hypertrophy and angiogenesis are closely integrated processes in cartilage and hypertrophy might contribute to angiogenesis [27, 28]. Therefore, induced hypertrophy and angiogenesis by TGFβ1 suggested TGFβ1 did not play a role in cartilage regeneration.

In osteoarthritis, chondrocytes undergo hypertrophic change and the ECM of the cartilage degenerated, followed by blood vessel invasion. Bone formation is often coupled with chondrocyte hypertrophy and angiogenesis in development. Of the top 10 most up- or down-regulated genes detected, COL10A1 and STC1 were related with chondrocyte hypertrophy and bone formation [29, 30]. SEPP1, which was closely associated with endothelial cells and were able to impair angiogenesis [31], was detected as one of the most down-regulated genes in this study. As TGFβ signaling pathway in endothelial progenitor cells would promote angiogenesis [32], it is possible that the signaling pathway played the same role in chondrocytes. Additionally, molecules related to blood vessel formation were up-regulated after TGFβ1 treatment. VEGF has possibly been involved in the pathogenesis of osteoarthritis [33] and our results provided evidence for its potential role in chondrocytes. Endothelial cell biomarkers, ESM1 and KDR were significantly up-regulated. Matrigel assay further showed that networks were formed by chondrocytes in the matrigel. According to our study, it could be speculated that *in vivo*, besides synovium and subchondral bone, angiogenic factors in the cartilage could also come from chondrocytes with TGFβ1 stimulation, which would promote the progression of OA through the assistance for blood vessel formation or invasion. However, as *in vitro* study cannot reflect the real situation *in vivo*, the functions and mechanisms of TGFβ1 on cartilage and chondrocytes need more explorations in the future.

In summary, the present study investigated the effect of TGFβ1 on human chondrocyte *in vitro* and found that angiogenic factors could be induced from chondrocytes upon TGFβ1 treatment. Therefore, chondrocytes with TGFβ1 stimulation might also play a role in angiogenesis in osteoarthritis. Although TGFβ1 are widely used to induce chondrogenesis in stem cells [34, 35], it seems it is not appropriate to use it for chondrocyte culture *in vitro* for practical use, especially for therapeutic purpose such as autologous chondrocyte implantation (ACI). It is speculated that high level of TGFβ1 might play a negative role in cartilage regeneration and chondrocyte culture. Therefore, usage of TGFβ1 for chondrocyte culture in clinic should be considered prudentially. Instead, targeting TGFβ1 or relevant receptors might be a strategy to prevent or alleviate progression of osteoarthritis.

## MATERIALS AND METHODS

### Isolation and culture of human articular chondrocytes

This study was approved by the Institutional Review Board of Shenzhen Second People’s Hospital and all experiments were performed in accordance with the relevant guidelines and regulations. Patients received detailed explanation of the study and submitted written informed consents. Human articular cartilage tissues were collected from knee joints of 7 patients who had undergone total knee replacement surgery. The cartilage samples were rinsed with phosphate buffer saline (PBS) plus penicillin and streptomycin (Gibco) for 3 times. The tissues were then cut to about 1 cubic millimeter and placed in 1mg/ml collagenase II (Sigma) for digestion at 37°C for 6 hours with shaking. After filtration and centrifugation, cells were resuspended with DMEM/F12 (Thermo Scientific Hyclone) plus 10% FBS (Gibco) and 1% non-essential amino acid (Sigma). The cells were seeded at the density of 4× 10^3^ cell/cm^2^ in 6-well plates. Media were refreshed every 3 days and the cells were passaged when the confluence reached 80% or above. TGFβ1 was added to the wells of plate when chondrocytes adhered to the bottom of the plate at different concentrations (2ng/ml, 5ng/ml and 10ng/ml). Cells with the confluence of 80% or above were collected and treated with Trizol reagent (Invitrogen) for total RNA isolation or fixed with 4% paraformaldehyde (PFA) for toluidine blue staining and immunocytochemical staining. Quantitative analysis of network formation was performed with Image J software and the data was presented as the means ± standard deviation from triplicate experiments.

### Cytochemistry and immunocytochemistry

The fixed cells were rinsed with PBS for 3 times and stained with 1% toluidine blue for 4h with shaking. The cells were then washed with water and observed. The images were captured using a Leica inverted microscope (Leica DM16000B).

The samples for immunocytochemical staining were first blocked with 10% normal horse serum for 1 hour at room temperature and incubated with primary antibody at 4°C overnight. Primary antibodies used in this study were anti-collagen type I (RD) and anti-collagen type II (Sigma). Secondary antibodies (Invitrogen) were applied on the second day after 3 washes of the samples with PBS. All the samples were mounted with the mounting solution Fluoroshield with DAPI (Sigma).

### Quantitative RT-PCR analysis

Total RNA was extracted from cells using Trizol reagent (Invitrogen) according to the manufacturer’s instructions and then was reversely transcribed using the Revert Aid First Strand cDNA Sythesis Kit (Fermentas). Quantitative RT-PCR was conducted with SYBR Green Supermix (BioRad) using aViiA 7™ Real-Time PCR Sytsem (Applied Biosystems). All samples were normalized to glyceraldehyde-3-phosphate dehydrogenase (GAPDH). Each assay was performed in triplicate. Primers used were listed in Table 4. The fold change in the expression was analyzed using the ΔΔCt method. Student’s t-test was used in independent data for statistical analysis. The differences were considered significant when the P value was<0.05.

**Table 4:** Primer sequences for quantitative PCR

Primers	Sequence 5′---- 3′	Information
hGAPDH-F	AAGGTGAAGGTCGGAGTCAA	GAPDH expression
hGAPDH-R	AATGAAGGGGTCATTGATGG	
hCol2A1-F	CTGTCCTTCGGTGTCAGGG	Col2A1 expression
hCol2A1-R	CGGCTTCCACACATCCTTAT	
hSMAD3-F	TCAACACCAAGTGCATCACC	SMAD3 expression
hSMAD3-R	CGGCAGTAGATGACATGAGG	
hSMAD7-F	CCAACTGCAGACTGTCCAGA	SMAD7 expression
hSMAD7-R	CCAGGCTCCAGAAGAAGTTG	
hCOL10A1-F	CATAAAAGGCCCACTACCCA	COL10A1 expression
hCOL10A1-R	GTGGACCAGGAGTACCTTGC	
hVEGFA-F	CAAGACAAGAAAATCCCTGTGG	VEGF expression
hVEGFA-R	GCTTGTCACATCTGCAAGTACG	
hESM1-F	TGTCAGCCTTCTAATGGGGA	ESM1 expression
hESM1-R	ACTGGCAGTTGCAGGTCTCT	
hKDR-F	CCTGTATGGAGGAGGAGGAA	KDR expression
hKDR-R	CGGCTCTTTCGCTTACTGTT	

### Western blot analysis

The proteins from different groups of cells were extracted by RIPA buffer (Cell Signaling) and denatured using Laemmli SDS sample buffer. The denatured samples were loaded onto a 12% gel, running at 90 V for 30min and 160V for 90min.The membranes carrying proteins were blocked with 5% non-fat dry milk (2.5g milk powder in 50ml TBST) for 1 hour at room temperature. Primary antibodies diluted in 5% non-fat dry milk (mouse anti-GAPDH (Sigma), 1:2000; goat anti-ESM1 (Santa Cruz), 1:1000) were applied to the membranes separately overnight with shaking at 4°C. Secondary antibodies (anti-mouse HRP, 1:3000, (Invitrogen); anti-goat HRP 1:2000, (Invitrogen)) were applied and incubated for 1 hour at room temperature the second day after washing the membrane with TBST for 3 times. ECL Western Blotting Detection Reagent (Pierce) and UVITEC Cambridge (UK) was used for the development. Densitometric analysis of Western blot was performed using ImageJ software. The integrated density was measured for 3 times and then used for statistical analysis.

### Flow cytometric analysis

Indirect immunofluorescence analysis was performed for chondrocytes with anti-ESM1 antibody (Santa Cruz) at 4 °C for 30 min. After two washes with wash buffer (PBS supplemented with 2% FBS), the cells were incubated with the secondary mouse anti-goat IgG-FITC antibody (Invitrogen) for 30 min in the dark. Following two more washes, the cells were resuspended in PBS and analyzed by flow cytometry (Beckman Coulter Navios). Cells incubated with secondary antibody only were used as the negative control.

### DNA microarray analysis

Affymetrix (Santa Clara, USA) Gene Chip Human Genome U133 Plus 2.0 Array was used in the study. This Array is widely used for human gene expression profile and allowed analysis of 47,000 transcripts. Samples from 3 patients were sent to CapitalBio Corporation (Beijing, China) for the experiment and the results were analyzed by Affymetrix Microarray Suite (MAS 5.0)[16, 17]. All the experiments of gene expression profiling were authorized to be performed by CapitalBio Corporation (Beijing, China). Briefly, cells treated or untreated (control) with 10ng/ml of TGFβ1 were harvested and total RNA was isolated with Trizol reagent (Invitrogen). Qualified RNA was used to synthesize a double-stranded cDNA that was used for the following biotin-tagged cDNA synthesis. The biotin-tagged cDNA was fragmented to strands with 35-200bp in length and then proceed to the hybridization process with Affymetrix Gene Chip Human Genome U133 Plus 2.0 Array. All the DEGs were analyzed with a free web-based Molecular Annotation System3.0 (MAS 3.0, www.capitalbio.com). Gene Ontology (GO) analysis and pathway analysis using Kyoto Encyclopedia of Genes and Genomes (KEGG) database were performed.

### Matrigel assay

Matrigel matrix (BD) was used for the tube formation assay. The matrix was melted at 4°C for at least 2 hours before the experiment and 50μL matrix was added in each well of the 96-well plate on ice. The plate was transferred to the incubator for about 30min to allow the matrix to gel. The cell density was adjusted to 1.2×10^5^ cell/mL and 100μL per well of the single cell suspensions was added into wells with gelled matrix. Each sample was examined in triplicate. The plate was then put into the incubator. Images were captured with an inverted microscope (Leica DMIL LED) 5 hours after the incubation. Angiogenesis inhibitor SFN 1.5uM (Sigma) was added to the control group. Three random areas for each sample were photographed. The total length of the capillary-like structures, the number of junctions and branches, and the total segments length were measured using ImageJ software and the data was presented as the means ± standard deviation from triplicate images.
